# 2D Digital Reconstruction of Asphalt Concrete Microstructure for Numerical Modeling Purposes

**DOI:** 10.3390/ma15165553

**Published:** 2022-08-12

**Authors:** Marek Klimczak, Irena Jaworska, Marcin Tekieli

**Affiliations:** Faculty of Civil Engineering, Cracow University of Technology, Warszawska 24 Street, 31-155 Cracow, Poland

**Keywords:** asphalt concrete, digital reconstruction, microstructure, finite element method, heat flow, plane strain

## Abstract

In this paper, we deal with the issue of asphalt concrete microstructure recognition for further numerical analysis. An efficient reconstruction of the underlying microstructure makes the composite analysis more reliable. We propose for this purpose a methodology based on the image processing and focus on a two-dimensional case (it can be easily used as a part of the 3D geometry reconstruction, however). Initially obtained geometry of the inclusions is further simplified to reduce the cost of the finite element mesh generation. Three straightforward geometry simplification algorithms are used to perform this process in a controlled way. Subsequently, we present the solutions of two problems, i.e., heat flow and elasticity (plane strain), in order to illustrate the effectiveness of the whole elaborated methodology. The numerical results were obtained using the finite element method. Consequently, an error analysis is demonstrated in order to refer the overkill mesh solutions to the ones presented in this study. The main finding of this paper is the efficient methodology dedicated to a digital reconstruction of the asphalt concrete microstructure by the image processing. It can be also extended to other materials exhibiting similar microstructure.

## 1. Introduction

### 1.1. Asphalt Concrete Microstructure Characterization

**Figure 1:**
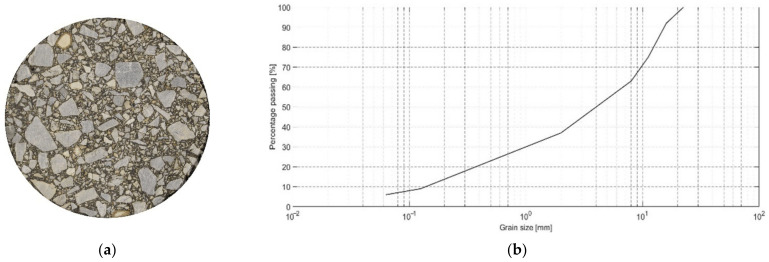
Top view of the cylindrical AC specimen (**a**) and the corresponding gradation curve (**b**).

Asphalt concrete (AC) is a popular composite with common applications in civil engineering. For instance, it is a typical material used for all bituminous layers of asphalt pavement structure [1,2,3,4]. AC is a compound of two main phases: mineral aggregate (more than 90% of the weight ratio) and a bituminous binder (remaining several percent of the ratio). Mineral aggregate comprises a number of aggregate fractions that can be illustrated with a gradation curve (see Figure 1). In order to improve the adhesion between aggregate particles and the binder, a very fine aggregate fraction (passing through the sieve of diameter equal to 63 µm) is additionally used. This aggregate fraction is called a filler. Some other additives are also applied for this purpose and due to other technological reasons. One can mention, among others, adhesive agents, fly ashes, and glass powder. In Figure 1, a typical AC gradation curve is shown. One can observe a similar weight ratio of each aggregate fraction, which is a characteristic property of the analyzed material. For a comprehensive description of the AC in the context of its numerical modeling, we refer the reader to [3].

In this study, we restrict ourselves to the reconstruction of the AC microstructure geometry in a biphasic mode, i.e., distinguishing only two main phases described above. This assumption is used due to the further numerical analysis limitation. It is infeasible, or at least prohibitively expensive, to account in the analysis for all aggregate fractions, particularly in the case of relatively large specimens and nonlinear material models of the constituents. A threshold value is typically used to specify the aggregate fractions that are geometrically reconstructed. In the majority of research papers, this value is equal to 2 mm [5,6,7,8]. Effective parameters of the asphalt binder (containing also the remaining small aggregate fractions) are used instead to compensate the aforementioned simplification. This phase is referred to as the mastic [7,8].

As the mineral aggregate used in AC is obtained via the crushing process, the boundaries of individual particles exhibit strong irregularities and need to be approximated. Similarly to the threshold value used for the minimum geometrically represented particle, a number of measures can be used to control this approximation (see Section 2). In this study, we do not analyze mechanisms that can be observed at lower scales and are also associated with the corresponding inclusions’ geometry at these levels. We do not analyze, for instance, the fact that open pores are infiltrated by the hot binder and a strong interface zone is created (cf. [8]). In numerical examples presented in Section 3, a perfect bonding is assumed.

The reconstruction of the AC microstructure is performed in a 2D space in this paper (cf. [1,8,9,10,11]). However, this methodology can be used in the extended form to reconstruct a 3D geometry as well. In general, a 2D AC microstructure representation is sufficient in some numerical analyses due to specimen geometry and applied boundary conditions (cf. [8,9,12,13]). The methodology presented in Section 2 is planned to be extended in our ongoing project to cover a 3D case. Reconstruction of the specimen microstructure in this space is performed on a sequence of “slices”. They can originate from mechanical treatment, but typically an X-ray-computed tomography is used for this purpose as non-destructive testing [5,8,14,15,16,17]. Thus, the methodology presented herein for a solely 2D case can be applied repeatedly for each of the aforementioned “slices” to reconstruct a full 3D microstructure geometry.

Below, we present several approaches to the AC microstructure geometry modeling. It should be stressed once again that this is also the focus of this study. The obtained numerical results (see Section 3) are to present the effectiveness of our framework. Thus, we concentrate in the next section on the geometry modeling. Additional information, referring, e.g., to the constitutive model used, are of the complimentary character. In this paper, we focus particularly on the AC microstructure geometry modeling, rather than on the whole pavement design process.

### 1.2. Numerical Representation of AC Microstructure

Accounting in numerical analysis for the AC microstructure geometry has been an active research field for decades [1,2,3,4,5,6,7,8,9,10,11,12,13,14,15,16,17,18,19]. The first numerical analysis of the asphalt pavement structure neglected the heterogeneity of the asphalt mix [10]. Instead, effective material parameters were phenomenologically obtained for each layer. Such a multi-layer domain can be analyzed numerically with much less computational effort. It is mainly due to a very simple geometry of the asphalt pavement structure itself. Disregarding the material microstructure enables one to generate a relatively coarse mesh in the finite element analysis (FEA); that is the most popular approach used in civil engineering. The aforementioned simplification in AC modeling is used even nowadays [18]. It is typically used when the focus of the research is on the effective AC material model rather than precise reconstruction of the complex geometry in the analysis.

The overall response of the AC to the external factors is highly affected by the mechanisms observed at the lower scales. In this paper, we restrict (both in the state-of-the-art description and in our analysis) to the microstructure, i.e., scale of a single aggregate particle. However, even examples of the modeling of selected mechanisms occurring at the much lower scale by the molecular dynamics can be found in the literature [20].

The first attempts of the AC microstructure modeling for the numerical analysis purposes were based on the geometry idealization. Selected applications of this approach, both for 2D and 3D space, are presented below.

Sepehr et al. demonstrated in [10] the necessity of the numerical AC analyses incorporating the microstructure geometrical modeling. Performing a number of plane strain computations and assuming the elastic behavior of all components, they studied dependencies of AC degree of compaction, binder stiffness, aggregate content, aggregate stiffness, aggregate size, and shape on the surface deflection. Importantly in the context of our study, the authors confirmed computationally that the aggregate shape influences the overall AC properties. A difference of about two percent in the maximum surface deflection was observed comparing aggregate particles with sharp irregular edges (representing crushed stone) and almost rounded particles (representing natural round aggregate). Due to the limitations of the computational resources at that time, the numerical results presented in [10] were obtained using very simplified shapes of the aggregate. There is also no detailed information on the procedure of the random microstructure geometry modeling.

The extension of the aforementioned research was presented in [17] by Liu et al. On the basis of the 3D True Sphericity parameter (3DTS), they investigated the response of the aggregate roundness on the AC sample response. The observations from [10] were confirmed and thoroughly analyzed in a 3D space. The procedure for the microstructure geometry modeling was much more advanced than that presented in [10] and is described in one of the proceeding paragraphs. The papers [10,17] are grouped together to emphasize the need for efficient microstructure geometry modeling in AC numerical analysis.

The latter can be considered as an independent research field. Thus, in a number of papers, one can find the idealized aggregate geometry [1,11,12,13,21]. The computational effort of the microstructure modeling is minimized in order to focus on other modeling aspects.

In [1], Kim et al. presented the multiscale framework for the asphalt pavement analysis. They investigated the quasi-static problem accounting for the material heterogeneity as well as the viscoelasticity and the anisotropic damage. The problem was solved in terms of the FE^2^ homogenization, as proposed by Feyel and Chaboche [22]. The whole methodology was illustrated first with a 2D academic problem of a tapered bar with a periodic microstructure. The aggregate particles were modeled as linear elastic and their geometry was idealized as hexagons of equal size. In the second example, the 2D representative volume element (RVE) accounted for much more realistic AC microstructure. The aggregate particles were modeled as irregular polygons. There was no information on the procedure of their generation, however.

The porous asphalt concrete (PAC) was studied in [11] by Mo et al. The focus was on the loss of the aggregate particles of the wearing course subject to moving wheel load (raveling). The analyzed pavement layer was idealized with a regular pattern of circular (2D) or spherical (3D) aggregate particles uniformly coated with a binder layer. An interfacial zone between the constituents was also analyzed. Behavior of aggregate and interface was assumed to be elastic, and the binder was modeled using the Burgers constitutive equations. Interface properties were assumed as the average value of the aggregate and binder properties (the elastic part of the Burgers model).

In [12], Mitra et al. simulated the Marshall test using the linear elastic model for the aggregate particles and the elastic-viscoplastic model for the binder mix. By the “binder mix”, the authors understand the mixture of the pure binder, air voids, and small rigid particles. The effective response of the binder mix is assessed using the stiffening factor approach. Thus, the only inputs to the developed model are the parameters of the aggregate particles, pure binder, and the volume fraction of the aggregate present in the binder mix. As far as the microstructure geometry representation is concerned, two approaches were used. Firstly, the so-called “synthetic microstructure” was generated with the aggregate particles modeled as non-overlapping ellipses with random location and orientation. Size distribution of the ellipses was constrained with the assumed gradation curve. Secondly, the actual microstructure obtained by the image processing of the X-ray-computed tomography scan was used. The authors did not develop the latter approach, but rather used the acquired data. Their conclusion was that there is no significant difference in results obtained using synthetic and actual samples. They remark that the analyzed problem could be specific, and they emphasize the need for efficient methods for actual AC microstructure geometry reconstruction.

Sadd et al. investigated in [13] the AC response to subject load with a specific setting. Namely, the aggregate particles were suggested to be modeled as rigid bodies linked with frame-like elements. The analysis was performed using the finite element method and its key point was the novel definition of the stiffness matrix representing the behavior of the binder mix. In fact, the framework was tested on a polyurethane matrix with a number of elliptical aluminum particles embedded.

Aigner et al. presented in [21] the upscaling scheme for the AC analysis with consideration of a number of analysis scales. Subsequent upscaling steps are performed using the continuum micromechanics approach. By means of the localization tensor **A**, effective parameters are assessed in terms of the lower scale quantities. The Mori-Tanaka scheme [23] is developed for this purpose. Aggregate particles of the idealized shape (ellipse) were used due to the known analytical formulas for the localization tensor **A**. At each step of the upscaling, the effective matrix parameters were assessed in terms of the underlying matrix-inclusion material. For the viscoelastic parameters, the corresponding principle was used. Laplace-Carson transform was used to compute effective creep functions, and its inverse was employed to express them in the time domain.

Nowadays, the most reliable method for the AC microstructure geometry recognition is based on the image processing that is mostly performed on the X-ray-computed tomography (XRCT) scans. One can find a number of such applications in the literature [5,8,12,14,15,16,17]. This method is developed in this study and is described in the next section. One should remark that this approach may lead to impractical microstructure geometry for the numerical analysis purposes. For example, in [15], reconstruction on the microstructure geometry for a cube with dimensions 24.5 × 24.5 × 24.5 mm^3^ required 1000^3^ voxels. In terms of the FEA, it would result in a problem solved using the same number of finite elements, which is the overkill mesh.

The synthetic microstructures are generated instead to model the AC microstructure geometry in a possibly reliable way. This approach is typically based on the Voronoi tessellation [7,15,18] or the Boolean operations on the geometry primitives [9]. Such methods deliver a microstructure geometry that is much simpler that the actual one obtained by the XRCT. Consequently, it does not require FE meshes of the prohibitively expensive density.

The paper is organized as follows. Section 2.1 describes sample and image preparation. Section 2.2 is devoted to the image processing performed on the AC specimen images. Section 2.3 presents the methodology for the controlled microstructure geometry simplification. In Section 3, numerical results for the selected AC images are presented. A discussion of the results is provided in Section 4.

## 2. Materials and Methods

### 2.1. Samples and Images Preparation

Specimens used in this study were obtained via drilling from the existing federal road near Cracow, Poland. They are cylinders, with a diameter base equal to 16 cm and a different height depending on the pavement structure present. For the purposes of this study, the specimens were cut into a number of 5 cm-thick slices with the circular saw using a sliding table in a laboratory of the Chair of Highway and Traffic Engineering of Civil Engineering Faculty (Cracow University of Technology, Kraków, Poland). Before further proceeding, the specimens were dusted off and treated with isopropyl alcohol in order to reduce the possible image quality deterioration.

**Figure 2:**
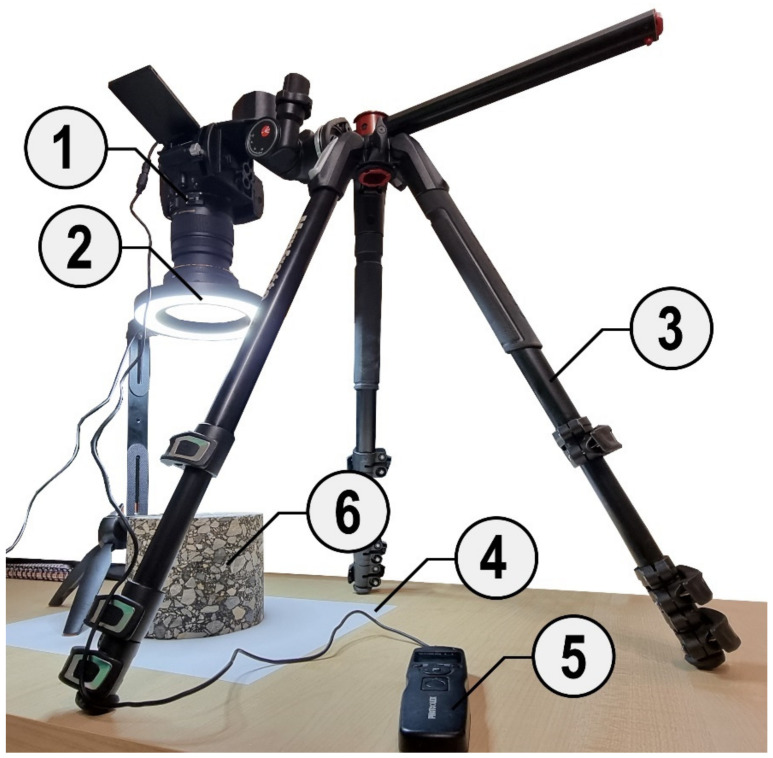
Test stand with DSLR camera (1), light source (2), tripod (3), uniform background (4), intervalometer (5) and tested specimen (6).

In order to ensure high-quality photos, which are the basis for further processing, the photos were taken with a digital single lens camera (DSLR) Nikon D5300 equipped with a high-quality lens Sigma 17–50 mm f/2.8 EX DC OS HSM with negligible radial distortion in order to ensure the best representation of the sample surface and the shape of grains both in its center and edges. The apparatus was placed on a stable tripod and directed perpendicularly to the sample surface. The photos were taken with a focal length of about 25 mm and an ISO-100 value. The exposure speed was set in the range from 1/50 to 1/25 s. Photos were taken with a resolution of 24 Mpx (6000 × 4000 px) and saved in a Nikon electronic format (NEF), which is a raw data storage format. In order to ensure the appropriate quality (high resolution and sharpness) of the photo, it was necessary to use sufficiently strong light with the appropriate color temperature. To avoid discoloration related to the color temperature of the light, LED light with a temperature range of 4000–5700 K was used, which is neutral towards the cold. A cable interval timer was used to trigger the shutter to avoid vibrations and to increase the sharpness of the photo. The test stand for taking photos of samples is shown in Figure 2. 

The samples were placed on a uniform white surface, which facilitated the separation of their proper, tested surface from the background. Prior to further processing steps, the top surface of the sample was constrained to a circular shape with background removal.

As the authors assumed that making the best quality photos would bring benefits at further stages of their processing and would increase the effectiveness of the image processing methods used, in order to sharpen the photo as much as possible, a calibration pattern was placed on the sample before each photo was taken. The pattern consisted of elements in the form of lines and circles contrasting with the background, which facilitated the proper setting of the lens. This was always done manually instead of the automatic mode in order to avoid the negative effect of back or front focus.

**Figure 3:**
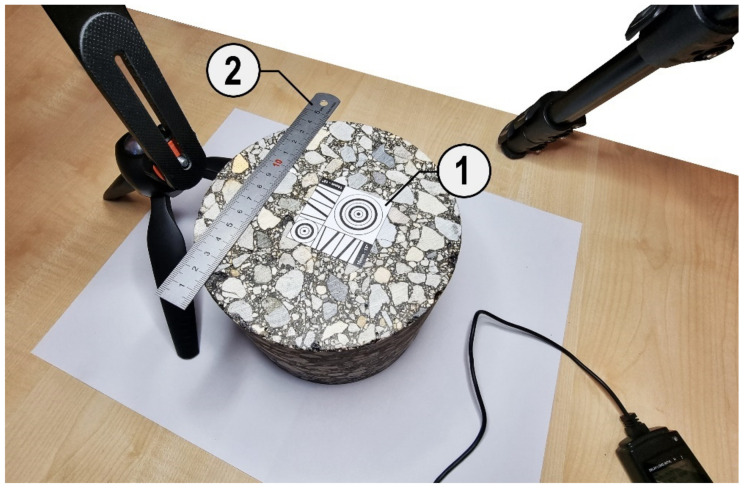
Specimen surface with the calibration pattern (1) and ruler as a length standard (2).

Additionally, to ensure the possibility of estimating the real size of grains expressed in millimeters, a length standard in the form of a ruler was placed on the surface of the sample. In this case, two photos were taken, one with and one without a reference, to ensure that the surface of the entire sample was visualized. Having a photo of a sample on which an object of known real length has been placed, it is possible to convert values from pixels to millimeters. This can be achieved simply by reading the length of the reference object in pixels in the photo, then comparing this value with the actual value in millimeters and determining the size of a single pixel in millimeters. Given the size of a single pixel in millimeters, it is possible to determine the actual size of any object in a photo based on its dimensions in pixels, assuming that we did not exceed the sharpening area associated with the sample surface. A fragment of the test stand with the elements described above is shown in Figure 3.

### 2.2. Image Processing

An image created according to the methodology described in the previous section is the input to the next step of microstructure geometry reconstruction. The final outputs of the image processing are the sets of pixel numbers (or indices) that identify the boundaries of every individual aggregate particle. The steps presented in the next sections are mostly standard in the object analysis performed using the image processing. However, they had to be adopted to the AC microstructure recognition.

#### 2.2.1. Conversion RGB to Grayscale Image

**Figure 4:**
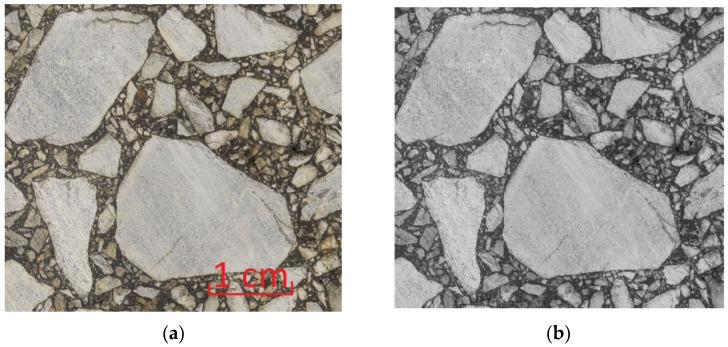
Truecolor AC specimen image with a length scale marked (**a**) and the corresponding grayscale image (**b**). The processed images are presented at the same length scale.

An image of the AC specimen is the truecolor image specified as the *m* × *n* × 3 matrix—*m* and *n* indices denote the image dimensions given in pixels, whereas the third number refers to three primary color components (red, green, and blue) used in the additive manner to reproduce other colors. The conversion to a grayscale image is performed to prepare the image for the further processing. The proceeding operations are tailored for the grayscale images that are specified as the *m* × *n* matrices. The entries store the information on the intensity (typically in the range [0, 1]). We compute the intensity (I) using the formula:

I = 0.2989 ∙ R + 0.5870 ∙ G + 0.1140 ∙ B(1)

where R, G, and B are the values of the red, green and blue components, respectively. The weights can be used arbitrarily, however. In the case of the aggregate made of the rock with a specific color, it can very profitable to modify these weights. It may significantly facilitate further image processing steps. Figure 4 presents an exemplary initial RGB image and its grayscale counterpart.

#### 2.2.2. Image Binarization

In our study, we assume the biphasic AC microstructure. Namely, we distinguish only the matrix and the inclusions. By the matrix we understand the binder mix, i.e., the binder and aggregate fractions smaller than a selected size. Remaining aggregate fractions are geometrically reconstructed as the inclusions. First, a grayscale image needs to be binarized for this purpose.

Binarization is based on a modification of the *m* × *n* matrix filled with intensity values to the *m* × *n* matrix filled with 0 s and 1 s only. The values below a specified threshold are substituted with 0 s and the remaining ones with 1 s. Contrary to the global thresholding based on a single value for the whole image, the adaptive thresholding is used in our research. A threshold is computed locally in a selected *i*-th pixel neighborhood.

**Figure 5:**
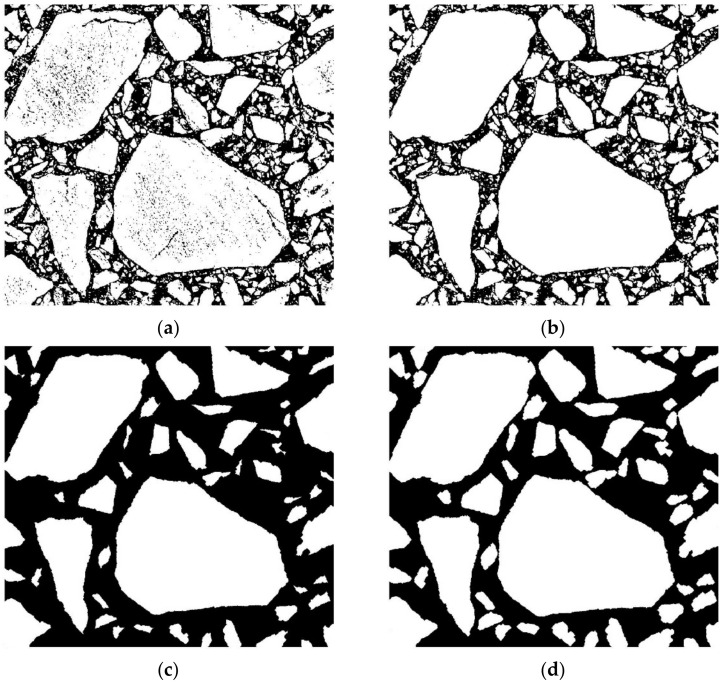
Binarized AC specimen image (**a**), processed image with filled inclusions (**b**), processed image after erosion and filtering operations (**c**), and final image after dilation operation (**d**).

The result of the binarization process for the image presented in the previous section (Figure 4) is shown in Figure 5.

Subsequently, the inclusions are processed in order to not contain any small objects that either are the numerical artifacts or are negligible in the numerical analysis (see Figure 5b). Then, the erosion operation is performed to eliminate (or at least substantially reduce) the number of adjacent inclusions. In the next step, we filter the inclusions to keep only those with sizes greater than a selected value (see Figure 5c). Finally, we perform the dilation process in order to compensate the diminished sizes of the previously eroded inclusions (see Figure 5d).

#### 2.2.3. Enhanced Binarization

In order to enhance the binarization process, we propose a simple algorithm described briefly below. Assuming that the best matrix-inclusion recognition would be obtained by the manual processing of every single inclusion, we proceeded as follows:

- An image is processed as described in Section 2.2.1 and Section 2.2.2 using the human visual inspection to control the process;
- A small number of inclusions is manually reconstructed;
- Those manually reconstructed inclusions are used as references;
- A threshold value described in Section 2.2.2 is iteratively updated to provide the improved agreement between the reference inclusion shapes and those reconstructed by the algorithm. So far, we have used the area of the inclusion as the quantity to be compared.

This simple algorithm can additionally improve the accuracy of the image processing steps presented in Section 2.2.1 and Section 2.2.2 performed purely on the basis of the human visual inspection.

### 2.3. Controlled Geometry Simplification

#### 2.3.1. Reference AC Microstructure

**Figure 6:**
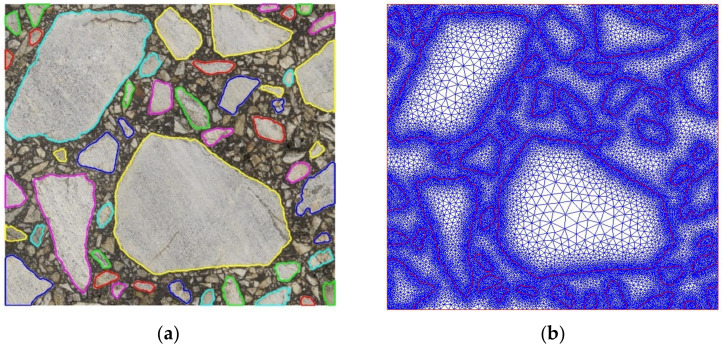
Boundaries of the captured inclusions overlaid on the original image (**a**) and the corresponding fine mesh (**b**).

For further comparison purposes, we generated a reference AC microstructure. It was created on the basis of the processed image shown in Figure 5d. The boundaries of all inclusions were captured as presented in Figure 6a. After this step, we computed the physical coordinates of inclusion vertices by the scaling operation. Precisely, we associated the center of every boundary pixel with its specified physical coordinates. Only the pixels along the whole domain boundary were treated in a different way. Namely, we shifted these vertices to the boundary in order not to trim the image.

Given the geometry of every single inclusion within the analyzed domain, one can proceed with a mesh generation. Herein, we used only triangular finite elements. An exemplary mesh generated for the inclusions captured according to the steps presented in Section 2.2 is shown in Figure 6b. A very high mesh density can be observed along the inclusion boundaries. This is due to the fact that its every pixel is transformed in a straightforward way to the corresponding geometry vertex. For this particular image, we obtained a so-called overkill mesh with about 220 thousand triangular finite elements and 110 thousand nodes. For the real-life applications, such a mesh would be prohibitively expensive and impractical for the numerical analysis. In this study, it is used only as the reference for further results. The microstructure geometry represented by the inclusion boundaries (shown in Figure 6a) is a starting point for its controlled simplifications.

Up to this step, our goal was to present the microstructure geometry recognition tailored for asphalt concrete and materials of a similar type. Any other efficient approach can also lead us to this point. The next sections are oriented on the effective geometry simplifications.

#### 2.3.2. Shortest Edge Elimination

The first of the proposed algorithms with reference to a single inclusion can be schematically described as follows:

- Reduce the initial number of vertices by simple removal of their specified percentage (10%, 20%, etc.). The vertices are removed with regular interval, i.e., the 1st, 11th, 21st, etc. (when the percentage equal to 10% is specified). This simplification is justified by the high resolution of the processed image.
- Remove iteratively the shortest edge along the inclusion boundary.

**Figure 7:**
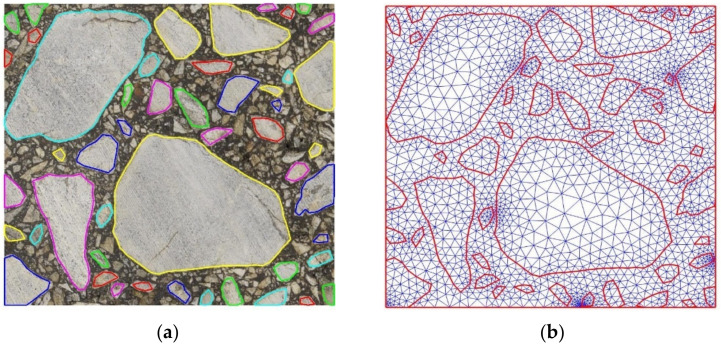
Boundaries of the simplified inclusions overlaid on the original image (**a**) and the corresponding fine mesh (**b**).

The simplified microstructure obtained using this straightforward algorithm is shown in Figure 7a. The reduction of vertices was selected at the extreme level of 90%. However, we left all the inclusion vertices along the domain edges in order not to introduce additional modeling error. Thus, the effective reduction is slightly below this number. Only two iterations of the algorithm were used. The resultant finite element mesh is presented in Figure 7b. The number of nodes is equal to about 5500 and the number of elements is equal to about 10,800.

#### 2.3.3. Local Geometry Enhancement

**Figure 8:**
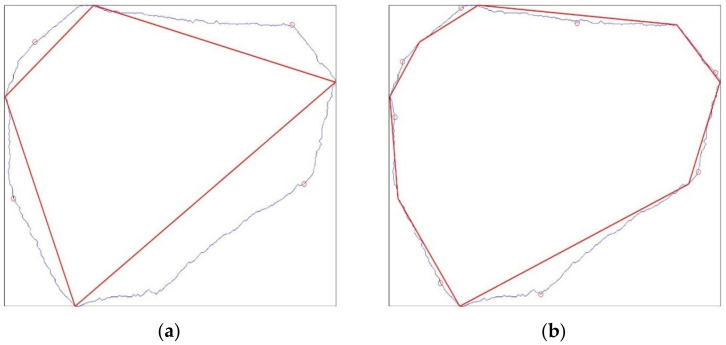

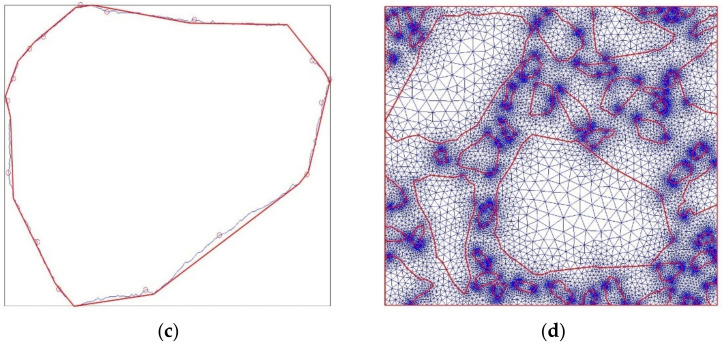
Specification of the outermost boundary pixels and the corresponding initial approximated inclusion geometry (**a**). Results of 2 subsequent iterations of the algorithm (**b**–**d**) the fine mesh obtained for the last approximated geometry. In (**a**,**c**), the light blue curve denotes the initial inclusion boundary and the red one shows its approximated counterpart. A single small red circle denotes the pixel from the boundary with the largest distance from the corresponding edge of the approximated geometry. In the next iteration, this point becomes a new vertex.

The second of the proposed algorithms is a kind of a simple interpolation. At the limit, it restores the initial boundary of the inclusion. Unlike the algorithm presented in Section 2.3.1, it starts with a very small number of boundary pixels (four is the initial maximum number) and selects additional pixels to be added. Schematically, the algorithm can be described as follows:

- Find the extreme (outermost) pixels along the inclusion boundary in all four directions, i.e., top, bottom, left and right (see Figure 8a). In the case of multiple values, we selected higher (for left and right extrema) and located more to the left pixels (for top and bottom extrema). Typically, a quadrilateral is generated.
- Iteratively, we looped over all the approximated geometry edges. The inclusion boundary pixel along the respective segment (see Figure 8b,d) with the largest distance from the approximated edge was searched. Consequently, a new vertex for the approximated geometry was added and the number of its edges increases. At this step, one can introduce additional requirements on the newly created edges. For instance, a minimum edge length can be verified before the current edge splitting.

The approximated microstructure and the corresponding fine mesh are shown in Figure 8d. The number of nodes is approximately equal to 10,500, and the number of triangular finite elements is slightly above 20,500. One can easily observe some subdomains with an extremely fine discretization. They are mostly located close to some short geometry edges. This effect can be minimized using the algorithm presented in Section 2.3.1.

#### 2.3.4. Convex Subdomain Approach

Another algorithm is based on the convex subdomains [24] created for each inclusion of the microstructure. The original boundary of the inclusion (set of pixels) is simplified and approximated by the convex hull. The set of pixels defines the input points and outputs the indices of the points that lie on the boundary of the convex polygon. In general, such a polygon is an intersection of all convex subdomains containing initial points. There are several classical algorithms [25] for computing the counterclockwise (usually) sorted sequence of extreme vertices for the searched convex envelope.

**Figure 9:**
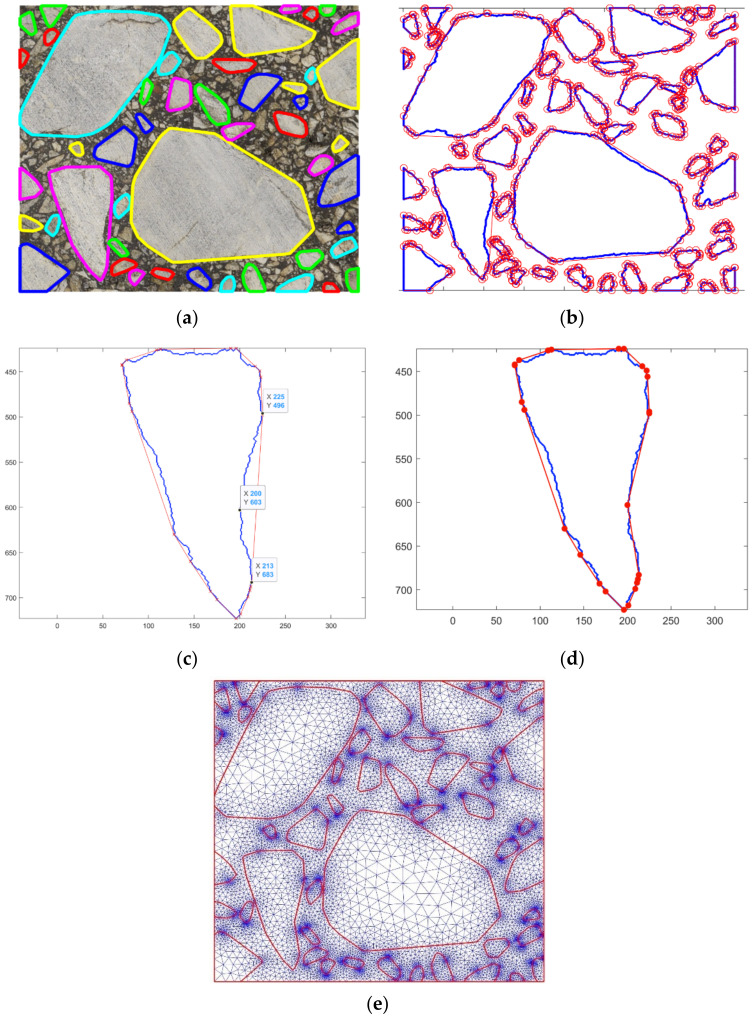
Boundaries of the approximated inclusions (initial set of 11,000 pixels) obtained by convex polygons (**a**) and their 860 vertices (**b**). Badly fitted convex area (**c**) corrected by inserting the additional vertex (**d**) and the fine mesh (**e**) corresponding to (**b**)—including correction shown in (**d**).

The approach was applied to the set above 11 thousand boundary pixels presented in Figure 6a. The obtained convex hulls consist of 860 vertices (Figure 9a,b). As it can be seen in the lower-left corner, due to the convexity, the edges of the approximated inclusions may overlap each other. In this case, an additional vertex (the boundary pixel with the largest distance from the approximated edge) has to be added to the most mismatched segment (Figure 9c,d). Additional nodes can also be added to edges that are too long for a better fit and a smoother transition between zones of different mesh densities. Conversely, for the boundary segments with high vertices density, the nodes can be removed using the approach outlined in Section 2.3.2.

For the particular microstructure shown in Figure 9b, a corresponding fine mesh was generated. The number of nodes was equal to about 16,200 and the number of triangular finite elements was slightly above 32,100. A very high mesh density can be observed in some subdomains. The geometry shown in Figure 8d can also be simplified using the algorithm presented in Section 2.3.2.

## 3. Results

In order to illustrate the effectiveness of the whole proposed methodology, we solved two simple 2D test problems: a stationary heat flow problem and an elasticity problem (with the plane strain assumed). They are both very important in the case of AC analysis. Herein, we present them as the independent problems; typically they are coupled to capture the overall thermo-mechanical AC response (cf. [26]). As the exact solutions of the two abovementioned problems are not given, we used the overkill mesh solutions obtained using the mesh presented in Figure 6b as the reference.

Before the numerical results are presented and compared with the reference solutions, one general remark is necessary. In Section 2.3.2, Section 2.3.3 and Section 2.3.4, we presented three straightforward algorithms dedicated to the simplification of the inclusion geometry. All of them guarantee the possibility to control this process. The first algorithm starts with the geometry very close to the initial one (obtained as described in Section 2.3.1) and introduces further simplifications. Contrarily, the remaining two algorithms begin with a very simple geometry and consequently modify it to perform a better approximation. All three algorithms can have a number of user-defined conditions specified in order to control the most important quantities of interest. Typical geometrical measures that can control this approximation process are: area, perimeter, curvature, and others.

In a loop over all inclusions, one can iteratively proceed with the subsequent geometry modification step provided that the “difference”, i.e. the differences in all the analyzed quantities of interest, between the initial and present inclusion geometry is above the specified tolerance. Specifying a very small tolerance, we are closer to the initial geometry, which captures all local oscillations. At this level, a trade-off between the precision of the microstructure geometry recognition and the finite element mesh density needs to be found. This decision is typically made on the basis of the available computational resources. Thus, the numerical results presented in this section should be considered only as the exemplary ones.

We began with the high-resolution image (see Figure 4a) and followed the methodology demonstrated in Section 2.2 to obtain the initial boundaries of all inclusions. Then, we modified them using all three algorithms presented in Section 2.3.2, Section 2.3.3 and Section 2.3.4. For the illustrative purposes, we present the results obtained using some arbitrary values for the number of iterations. The numbers of resulting triangular finite elements are compared with the overkill mesh. Consequently, the modeling error introduced by the microstructure geometry simplification is measured for the heat flow and elasticity problems. It should be stressed, however, that all three algorithms can produce similar microstructures. The focus is rather on the dependence of the general geometry simplification on the results. The pros and cons of the algorithms themselves are discussed in Section 3.3.

### 3.1. Heat Flow Problem

**Figure 10:**
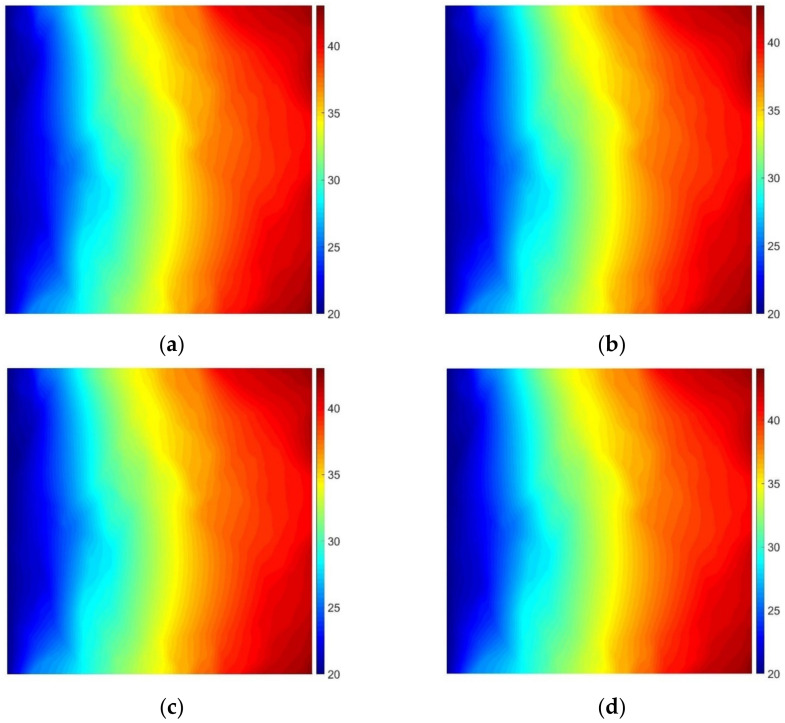
Map of temperature [°C] obtained using the overkill mesh (**a**) and meshes corresponding to the approximated geometry using shortest edge elimination (**b**), local geometry enhancement (**c**), and convex subdomain approach (**d**).

In this example, we analyze a square AC sample of dimensions 4 cm × 4 cm illustrated with Figure 4a. At the left edge, a constant temperature of 20 °C is assumed. The remaining edges are heated with the intensity of 20 W/m. Thermal conductivity is equal to 0.85 W/(mK) for the aggregate (inclusions) and 4.5 W/(mK) for the asphalt binder (matrix), respectively. The distribution of temperatures obtained for the reference fine mesh and for the meshes corresponding to the simplified geometry is shown in Figure 10. As far as the geometry simplification is concerned, we used the meshes referred to:

- in Figure 6b, which was generated on the basis of the initial microstructure geometry without any simplification,
- in Figure 7b, which was generated using the shortest edge elimination algorithm with the percentage of eliminated boundary pixels equal to about 90%,
- in Figure 8d, which was obtained after 2 local geometry enhancements, and
- in Figure 9e, after geometry correction.

Comparing the results shown in Figure 10 is almost impossible only with the human eye inspection. Instead, we provide in Table 1 a short comparison of the number of degrees of freedom (NDOF) used for each algorithm and the maximum temperature computed for the domain. Both the NDOF reduction and the error are related to the reference mesh/solution, i.e., we computed the NDOF reduction as

(NDOF_REF_ − NDOF_H_)/NDOFREF × 100% (2)

where NDOF_REF_ stands for the overkill mesh number degrees of freedom and NDOF_H_ is the number of degrees of freedom of the solution obtained using a coarser mesh resulting from the simplified geometry. Consequently, the solution error is computed as

|U_MAX,REF_ − U_MAX,H_|/|U_MAX,REF_| × 100% (3)

where U_MAX,REF_ denotes the maximum solution value in the analyzed domain (herein, it is the temperature) obtained using the overkill mesh. U_MAX,H_ is the maximum solution value obtained using a respective coarser mesh.

One can observe a very good agreement of the coarser meshes’ solutions with the reference one. The relative error computed according to (3) does not exceed 2.5% with the simultaneous reduction of the NDOF approximately equal to one order of magnitude (85.30–95.00%).

### 3.2. Linear Elasticity (Plane Strain) Problem

In this example, we analyze a square domain of dimensions specified in the previous section. Homogeneous Dirichlet boundary conditions (displacements equal to zero) are assumed at the left edge, and the uniformly distributed load of intensity equal to 80 kN/m is applied along the right edge to simulate the compression process of the AC specimen. The remaining edges are not subject to any load. The Poisson ratio for the aggregate particles and for the asphalt binder is equal to 0.3. There is a large difference in the Young moduli, however. This parameter is equal to 90 GPa for the inclusions and 10 GPa for the bituminous matrix.

As in the previous example, we solved this problem using the reference fine mesh and three coarser meshes. All of the meshes were the same as those used for the heat flow problem.

**Figure 11:**
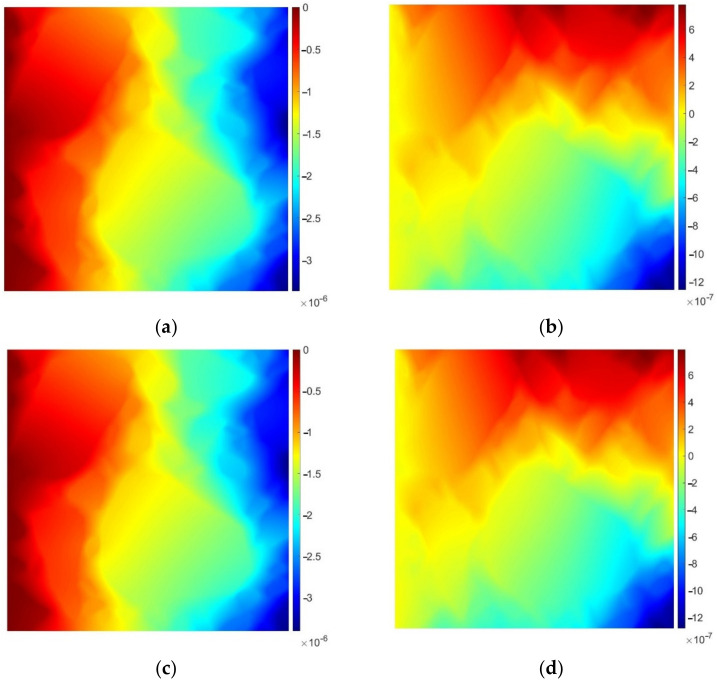

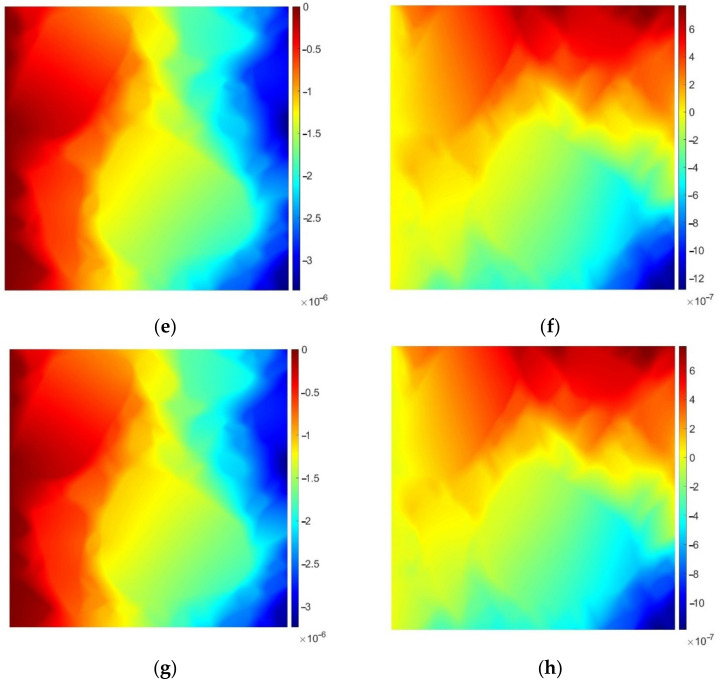
Horizontal (**a**,**c**,**e**,**g**) and vertical (**b**,**d**,**f**,**h**) displacement components [m]. The first row presents the reference solution and the remaining ones refer to the geometries obtained using the shortest edge elimination, the local geometry enhancement and the convex subdomain approach, consecutively.

In Figure 11, we present the horizontal (left column) and vertical (right column) displacement components obtained using all four meshes.

A very good agreement between the reference solution and the ones obtained using the coarser meshes can be observed also for the linear elasticity problem. In Table 2, the NDOF reduction and the solution error are presented. Since we use the same meshes as for the heat flow problem, the NDOFs for the plane strain are simply the doubled values of those presented in Table 1. Consequently, the NDOF reduction is the same as in the previous example. The solution error is computed according to (3). Instead of the maximum value used for the scalar field (temperature), we use the magnitudes of the displacements (square root of u_x_^2^ + u_y_^2^, where u_x_ and u_y_ stand for the horizontal and vertical displacement component, respectively).

Also in this example, the solution error was kept at the acceptable level. It does not exceed 4% for any of the geometries obtained using the algorithms described in Section 2.3.

### 3.3. Discussion

In this paper, we proposed the methodology for the 2D digital reconstruction of the AC microstructure for the numerical modeling purposes. Starting with high-quality truecolor images, we obtained the image processing boundaries of the aggregate particles. A threshold value was specified in order to limit the size of the fractions of interest, which is a typical operation in the AC numerical modeling. Subsequently, we proposed three straightforward algorithms for the aforementioned boundaries’ simplification. They can fully reconstruct the initial boundary and enable control of the geometry simplification process. One can build-in a number of conditions to be verified before the next simplification step/iteration. Thus, the geometrical measures that are the most suitable for the analyzed geometry can be used. One of our future research efforts is to propose a set of such geometrical measures that are well-suited for the AC microstructure.

The proposed methodology was illustrated on two test problems, i.e., stationary heat flow and linear elasticity. For both tests, the solutions obtained for the simplified inclusion geometries were in very good agreement with the reference ones. For the illustrative purposes, we kept various simplification levels, which resulted in approximately 85–95% reduction of the NDOF. The solution errors did not exceed 5%, which demonstrates the applicability of the introduced methodology.

The main finding of this research is the overall methodology, which enables a reliable numerical modeling of asphalt concrete. Herein, we illustrated our approach on numerical tests performed on a relatively small specimen. However, this approach can be particularly profitable in the case of whole asphalt pavement modeling with the consideration of the layers’ microstructure. It should be stressed that the computational cost of the geometry modification is paid only once at the preprocessing stage. Substantial decrease of the NDOF resulting from this modification reduces the computational time of possible transient and nonlinear analyses. Importantly, the user of the proposed methodology can easily control the trade-off between NDOF reduction and precision in microstructure recognition.

In summary, the main advantage of the developed methodology relies upon the simplification of the AC microstructure geometry resulting in further reduction of the NDOF. As it was demonstrated in the previous sections, the proposed simplifications do not introduce a large error to the solution compared to the reference solution. It should be noted, however, that our framework is not fully automatic. It is user-controlled, which means that the user is supposed to specify several threshold values. They influence the effectiveness of the whole framework, since their proper specification can be considered as a kind of bottleneck.

## 4. Conclusions

The main conclusions of this study are as follows:

- Image processing can be used in order to reconstruct the AC microstructure geometry.
- The initial inclusion boundaries can be effectively simplified using the algorithms presented in this paper.
- A large NDOF reduction can be obtained due to the user-controlled microstructure geometry simplification with a small solution error introduced.

## Figures and Tables

**Table 1:** Comparison of the results obtained for different algorithms (heat flow problem).

Algorithm	NDOF	Maximum Temperature [°C]	NDOF Reduction [%]	Relative Error [%]
Reference Solution	110,557	43.03	-	-
Shortest Edge Elimination	5524	42.72	95.00	0.72
Local Geometry Enhancement	10,445	43.01	90.55	0.05
Convex Subdomain Approach	16,251	44.09	85.30	2.46

**Table 2:** Comparison of the results obtained for different algorithms (linear elasticity problem).

Algorithm	NDOF	Maximum Displacement Magnitude [m]	NDOF Reduction [%]	Relative Error [%]
Reference Solution	221,114	3.37 × 10^−6^	-	-
Shortest Edge Elimination	11,048	3.41 × 10^−6^	95.00	1.19
Local Geometry Enhancement	20,890	3.36 × 10^−6^	90.55	0.30
Convex Subdomain Approach	32,502	3.24 × 10^−6^	85.30	3.86

## Data Availability

Not applicable.
